# Enhancing queries for code generation with reinforcement learning

**DOI:** 10.1038/s41598-025-21271-4

**Published:** 2025-10-24

**Authors:** Dawei Yuan, Guojun Liang, Tingting Li, Suping Liu

**Affiliations:** 1 School of Computer Science, Guangdong University of Science and Technology, Dongguan, 523083 China; 2 School of Information Technology, Halmstad University, Halmstad, 30118 Sweden

**Keywords:** Reinforcement learning, Code generation, Prompt engineering, Parameter-efficient fine-tuning, Engineering, Mathematics and computing

## Abstract

We present a reinforcement learning framework that enhances natural language queries to improve DeepSeek code generation. A parametric refiner (Qwen with LoRA) is trained via REINFORCE while the generator remains fixed, using a scalar reward that can combine text similarity (BLEU-4, ROUGE-L, F1, Overlap) with execution signals (unit tests, syntax/timeout penalties). On the DS1000 benchmark (800 train / 200 test), RL4QE improves the code similarity by 34.3%. Ablations show that BLEU-4 is the most reliable text reward overall (with F1 competitive on a larger scale), and LoRA with rank $r{=}8$ outperforms complete fine-tuning on most metrics while being more parameter efficient. The approach is transferred across foundation models (e.g., Qwen1.5/2/2.5 variants), where architecture often matters more than size. RL4QE is easy to integrate in practice (LoRA in attention projections) and supports reproducibility.

## Introduction

Large Language Models (LLMs) have transformed code generation and programming assistance^1^, with DeepSeek particularly excelling in logical reasoning and programming tasks^2^. However, several critical challenges persist in AI-driven programming: The first challenge is that the precise design of the query for optimal code generation remains difficult^3^. Although models produce syntactically correct code, outcomes heavily depend on input prompts, complicating systematic evaluation and enhancement. The second challenge is developing models to produce code that meets specific criteria, which remains problematic^4,5^. Current practices rely on manual prompt crafting, missing automated refinement opportunities. The third challenge is that the lack of robust training methods for incremental improvement in LLM makes optimization difficult^6–8^. Recent research addresses these through manual refinement and template-based methods^9–11^, prompt engineering, and chain-of-thought prompting^12–14^. However, these provide static, rather than adaptive, feedback-learning solutions. Existing query enhancement strategies are based on predetermined rules, failing to capture the intricate relationship between natural language and code^15,16^ and lacking systematic refinement based on the quality of generated code^17–19^. To address these challenges, we propose a reinforcement learning framework for query optimization with DeepSeek, using LoRA fine-tuning of Qwen to dynamically refine queries based on similarity between generated and reference code^12,20–27^. Through empirical analysis using DS1000^28^, we demonstrate significant quality improvements in programming tasks^2,29–33^, with extensive evaluation in programming contexts that establishes the foundations for future research^34–39^. The main contributions of this paper include:

- We propose an RL-based method to refine queries for DeepSeek code generation, learning from the results of generated code.
- We use a dual-model design: a learnable refiner (Qwen+LoRA) and a fixed generator (DeepSeek), with LoRA applied to attention projections for efficiency.
- We introduce a multi-aspect reward that combines text similarity (BLEU/ROUGE-L/F1/Overlap) and execution signals (unit tests, syntax penalty) to reflect practical code quality.

### Large language models for code generation and query enhancement

LLMs have advanced code generation from natural language to executable code^1–3,6^. Performance still depends heavily on query quality^5^. Earlier work improves queries via pseudo-relevance feedback, knowledge methods, and semantic parsing^24,40–42^, and addresses the *vocabulary problem*^17,18,26^. Recent LLM techniques add dense retrieval, rewriting, knowledge enhancement, and explanations^32,38,43,44^, with Self-RAG, CoT, and few shots prompting as strong baselines^12,15,16,20^. We follow this line, but make the refiner parametric and trainable with RL.

### Parameter-efficient fine-tuning (PEFT) with LoRA

PEFT reduces computation while preserving performance^20–23^. LoRA adds a low-rank update to frozen weights,

1 $$\begin{aligned} W = W_0 + AB, \end{aligned}$$

reducing trainable parameters and avoiding forgetting^26,27,29–33^. Previous work shows that LoRA maintains general language ability while adapting to code^35–39,41,42,44–47^. We apply LoRA to Qwen attention projections ($W_q,W_k,W_v,W_o$) for efficient query refinement^13,14,34,40,43,48–57^.

### Generalized preference automation (GPRO) for automated prompt optimization

GPRO frames prompt optimization as RL over prompts^1–12,15–33,35–37^. Our work differs in three ways: (1) a dual-model pipeline (learnable refiner + fixed generator), (2) LoRA-targeted attention for efficiency, and (3) the option to integrate execution-aware signals alongside text metrics. This design aims to adapt queries from outcome feedback rather than only template engineering.

### Reinforcement learning for LLM optimization

RL improves LLM beyond supervised learning through preference modeling and policy optimization^1,2,4,5,7–14,28,34,38–57^. We adopt a lightweight REINFORCE setup with a baseline and regularization of entropy for stability, focusing on training only the refiner while keeping the generator fixed, making the method practical for code generation scenarios.

## Methods

The DeepSeek-Chat and Qwen models were used as fixed generators and parametric refiners in this study. Their use was limited to generating code based on queries and refining queries based on rewards, respectively. They were not involved in the conceptualization, writing, or analysis of the manuscript.

**Figure 1:**
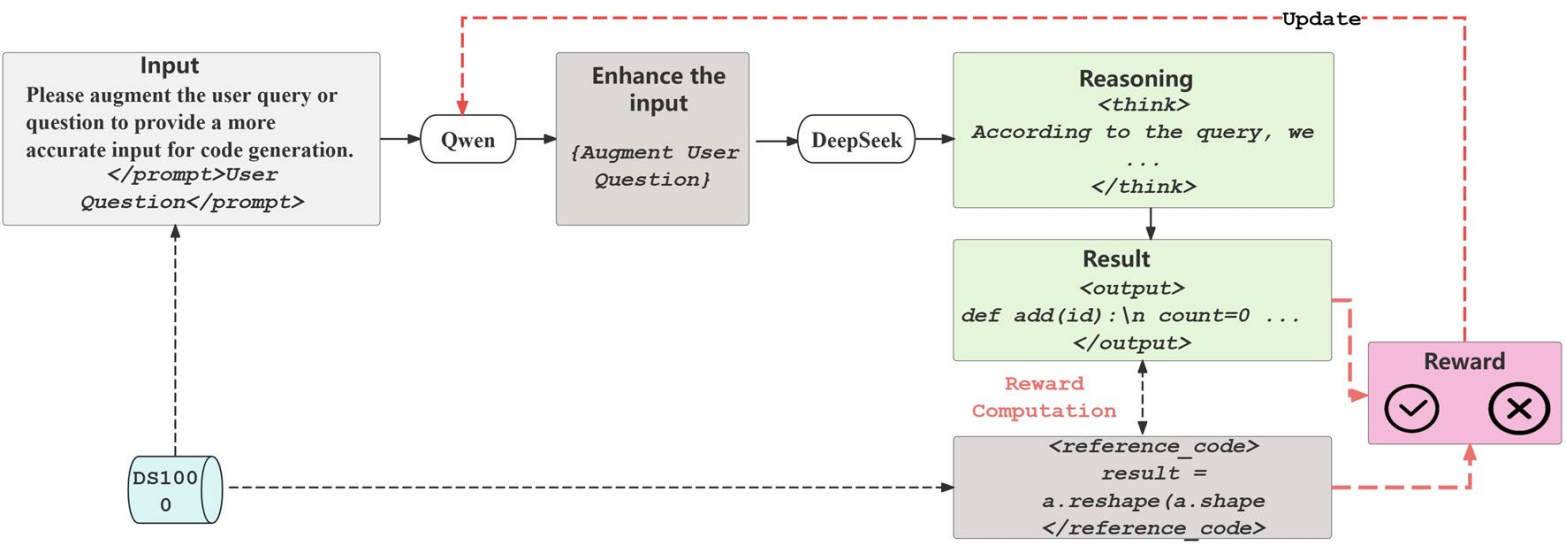
Overview of our approach.

**Figure 2:**
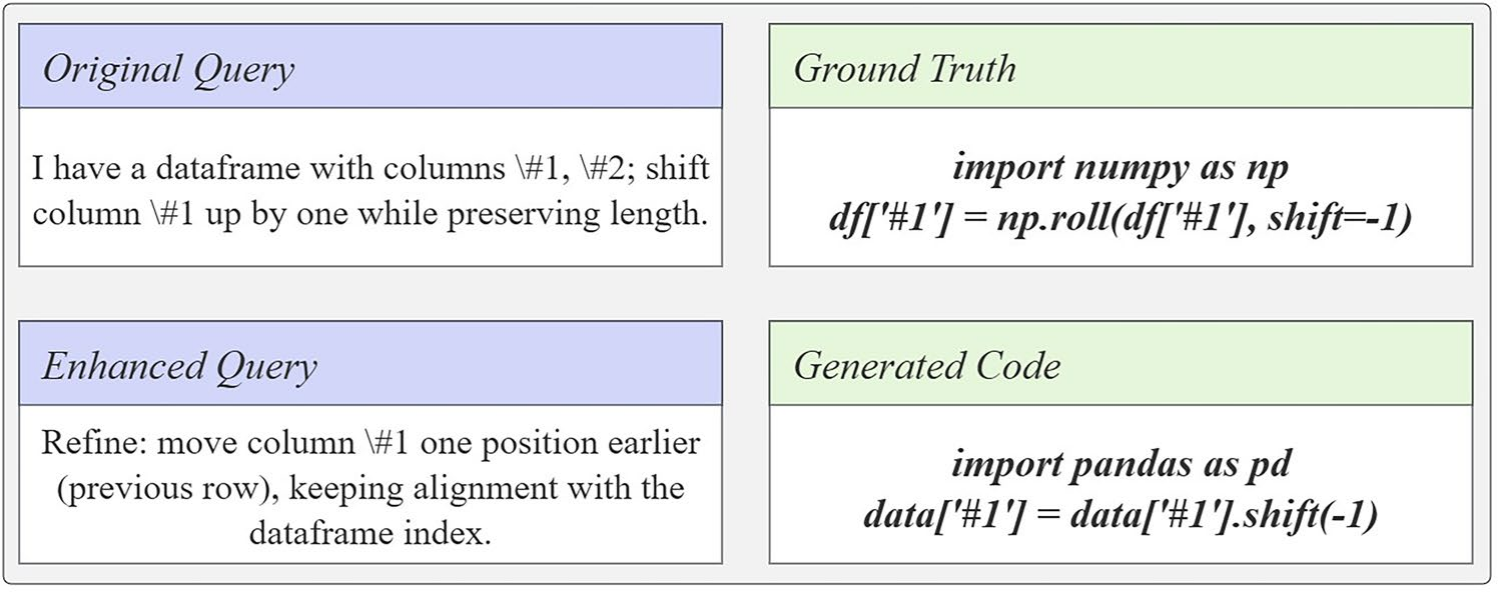
Query enhancement example showing original and enhanced queries alongside their corresponding code.

In this section, we introduce our reinforcement learning approach for query enhancement: we formalize the task, define the reward, and detail LoRA-based training on Qwen. Figure 1 outlines the pipeline: Qwen (with LoRA) refines the original query *q* in $q'$, DeepSeek generates code *c*, we compare *c* with reference $c^*$ to compute the reward *R*, and update the refiner. We use the following notation inline: *q* (original query), $q'$ (enhanced query), *c* (generated code), $c^*$ (reference code), *R* (reward); $W_q,W_k,W_v,W_o$ are attention projections, and LoRA applies a low-rank update $W = W_0 + AB$. Figure 2 shows a concrete case: The refined query yields a more precise code, evaluated against the ground truth via ROUGE-L.

### Problem formulation

We model query enhancement as a reinforcement learning problem. Given a query in natural language *q*, the refiner produces an enhanced query $q'$. Let $\mathscr {D}=\{(q_i,c_i^*)\}_{i=1}^N$ be queries with reference code $c^*$. We define a refiner $f_\theta : q \mapsto q'$ and a fixed generator $g: q' \mapsto c$. The goal is to learn $\theta$ to make *c* close to $c^*$ with the reward $R(c,c^*)$:

2 $$\begin{aligned} \theta ^* = \arg \max _\theta \,\mathbb {E}_{(q,c^*) \sim \mathscr {D}}\!\big [\,R\big (g(f_\theta (q)),\,c^*\big )\big ]. \end{aligned}$$

We implement $f_\theta$ with Qwen (full fine-tuning or LoRA) and train it by RL. We use REINFORCE with a learned baseline *b*(*q*) and regularization of entropy:

$$\mathscr {L}(\theta ) = -\,\mathbb {E}_{q'\sim f_\theta (\cdot |q)} \!\Big [(R(g(q'),c^*) - b(q)) \,\log f_\theta (q'|q)\Big ] \;-\; \lambda \, H\!\big (f_\theta (\cdot |q)\big ).$$

Rewards are normalized per batch to reduce variance. We optimize by gradient descent with the clip norm *1.0*: $\theta \leftarrow \theta - \alpha \nabla _\theta \mathscr {L}(\theta )$.

### Query enhancement

We study two ways to refine queries: full fine-tuning and LoRA. The complete fine-tuning updates all weights $W_0 \!\in \! \mathbb {R}^{d\times k}$ to *W*, but it is heavy and may forget pretraining. LoRA is efficient:

3 $$\begin{aligned} W = W_0 + \Delta W = W_0 + BA, \end{aligned}$$

where $B\!\in \!\mathbb {R}^{d\times r}$, $A\!\in \!\mathbb {R}^{r\times k}$, and $r \ll \min (d,k)$. We apply LoRA to attention projections ($W_q,W_k,W_v,W_o$). The refiner uses a simple template *p*(*q*) to transform *q* into $q'$. We compare our RL-based refiner with two strong baselines: Chain-of-Thought (CoT)^12^ and Retrieval-Augmented Generation (RAG)^58^. CoT modifies the prompts with hand-crafted reasoning; RAG augments the prompts with retrieved top-*k* snippets. Our method learns a parametric refiner (Qwen+LoRA) from outcome rewards, while the generator (DeepSeek) is fixed. In fairness, all methods use the same generator, decoding, and metrics (CSS, precision, recall, F1).

**Table 1:** Query enhancement methods.

Method	Learnable	Retrieval	Execution-aware	Change
CoT^12^	–	–	–	Prompt
RAG^58^	–	$\checkmark$	–	Prompt+Context
RL4QE	$\checkmark$	–	$\checkmark$	Refiner

Table 1 contrasts CoT and RAG, which modify prompts heuristically or through the retrieved context (nonlearnable, text only), with RL4QE, which trains a parametric refiner using outcome rewards and execution signals. Here, execution-aware denotes the use of execution outcomes (e.g., syntax / runtime errors, timeouts) as reward or guidance during training/evaluation. RL4QE integrates these signals and guides DeepSeek via LoRA on attention projections.

### Reward function

We define a scalar reward. For RQ1, we isolate the *text-only* rewards by running four separate settings (one per metric): Overlap, ROUGE-L, BLEU-4, and F1.

4 $$\begin{aligned} R(c,c^*) \;=\; \lambda _{\text {text}} \,\tilde{R}_{\text {text}}(c,c^*) \;+\; \lambda _{\text {exec}} \,\tilde{R}_{\text {exec}}(c), \end{aligned}$$

where $$\tilde{R}_{\text {text}}, \tilde{R}_{\text {exec}} \in [0,1]$$ and $$\lambda _{\text {text}}+\lambda _{\text {exec}}=1$$.

Overlap (token-set coverage of reference):

5 $$\begin{aligned} \textrm{Overlap}(c,c^*) \;=\; \frac{|\,\textrm{words}(c)\cap \textrm{words}(c^*)\,|}{|\,\textrm{words}(c^*)\,|}. \end{aligned}$$

ROUGE-L (LCS-based F-measure; higher is better):

6 $$\begin{aligned} P_{\text {lcs}}=\frac{\textrm{LCS}(c,c^*)}{|c|},\ \ R_{\text {lcs}}=\frac{\textrm{LCS}(c,c^*)}{|c^*|},\ \ \mathrm {ROUGE\text {-}L}=\frac{(1+\beta ^2)P_{\text {lcs}}R_{\text {lcs}}}{R_{\text {lcs}}+\beta ^2 P_{\text {lcs}}}. \end{aligned}$$

BLEU-4 (smooth 1–4 gram precision with brevity penalty):

7 $$\begin{aligned} \mathrm {BLEU\text {-}4} \;=\; \textrm{BP}\cdot \exp \!\Big (\sum _{n=1}^{4} w_n \log p_n\Big ),\quad \textrm{BP}=\min \!\big (1, e^{1-\frac{|c^*|}{|c|}}\big ). \end{aligned}$$

F1 (token-level overlap, order-agnostic):

8 $$\begin{aligned} \textrm{Precision}=\frac{|\,T_c\cap T_{c^*}\,|}{|\,T_c\,|}. \end{aligned}$$

9 $$\begin{aligned} \textrm{Recall}=\frac{|\,T_c\cap T_{c^*}\,|}{|\,T_{c^*}\,|}. \end{aligned}$$

10 $$\begin{aligned} \textrm{F1}=\frac{2\cdot \textrm{Precision}\cdot \textrm{Recall}}{\textrm{Precision}+\textrm{Recall}}. \end{aligned}$$

The text reward for a run is one of the above:

11 $$\begin{aligned} R_{\text {text}}(c,c^*) \in \{\textrm{Overlap},\ \mathrm {ROUGE\text {-}L},\ \mathrm {BLEU\text {-}4},\ \textrm{F1}\},\quad \tilde{R}_{\text {text}}=\textrm{Norm}\!\big (R_{\text {text}}\big )\in [0,1]. \end{aligned}$$

Execution-aware reward (not used in RQ1) combines unit test signals:

12 $$\begin{aligned} u \in [0,1]\ \text {(UT\%)},\quad s \in \{0,1\}\ \text {(syntax OK)},\quad t \in \{0,1\}\ \text {(timeout)}, \end{aligned}$$

13 $$\begin{aligned} R_{\text {exec}}(c)=u-\gamma _{\text {syn}}(1-s)-\gamma _{\text {to}}t,\quad \tilde{R}_{\text {exec}}=\textrm{Clip}_{[0,1]}\!\big (R_{\text {exec}}\big ). \end{aligned}$$

In RQ1, we set $\lambda _{\text {text}}{=}1,\ \lambda _{\text {exec}}{=}0$ and compare the four text rewards (one per run). In other experiments, we may include the execution feedback with $\lambda _{\text {exec}}>0$.

### Reinforcement learning

We train the refiner with REINFORCE. The pipeline is: $q \rightarrow q'$ (Qwen $f_\theta$) $\rightarrow c$ (DeepSeek *g*) $\rightarrow R(c,c^*)$ $\rightarrow$ update $\theta$. For efficiency, we separate evaluation (without gradients) and training (with gradients): in evaluation, we generate $q'$, *c*, and compute *R* under torch.no_grad(), then update $f_\theta$ using the stored $(q,q',R)$.

We use a learned baseline *b*(*q*) and regular entropy:

14 $$\begin{aligned} \mathscr {L}(\theta ) \;=\; -\,\mathbb {E}_{q'\sim f_\theta (\cdot |q)}\!\Big [\big (R(g(q'),c^*)-b(q)\big )\,\log f_\theta (q'|q)\Big ] \;-\; \lambda \, H\!\big (f_\theta (\cdot |q)\big ). \end{aligned}$$

Rewards are normalized per batch to reduce variance. We optimize by gradient descent with clip-norm 1.0 and optional gradient accumulation:

15 $$\begin{aligned} \theta \leftarrow \theta - \alpha \nabla _\theta \mathscr {L}(\theta ). \end{aligned}$$

The LoRA fine-tuning updates only the projections of the target attention ($W_q,W_k,W_v,W_o$), reducing memory. We keep the generator *g* fixed. The best checkpoint is selected by the validation reward.

### Algorithm

We train a refiner $f_\theta$ (Qwen) with rewards from the code generated by DeepSeek.

**Algorithm 1:**
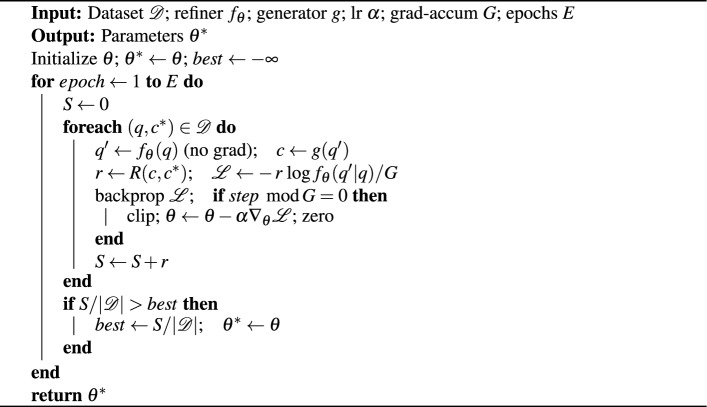
RL4QE: Reinforcement Learning for Query Enhancement

Algorithm 1 provides an overview of the training process, which involves evaluation to collect rewards without using gradients, followed by updating the refiner via REINFORCE incorporating entropy. This process employs gradient accumulation (*G*) and a clip norm of *1.0*. The optimal checkpoint is selected based on the average reward obtained during validation. The iterative cycle includes the refiner $f_\theta$ (Qwen), a fixed generator *g* (DeepSeek), and the reward *R*, integrating LoRA into the attention projections to improve efficiency.

## Results

### Research questions

We carried out a comprehensive series of experiments using the DS1000 dataset to assess the effectiveness of our reinforcement learning-driven query enhancement method for code generation, focusing on three critical research questions.

- RQ1: Which reward function design performs best for query enhancement?
- RQ2: How does LoRA fine-tuning compare to full fine-tuning?
- RQ3: How does our approach perform on different foundation models?

### Experimental setting

**Table 2:** Experimental configuration and dataset information.

Category	Component	Specification
Models	Query Enhancement (Base)	Qwen-7B
	LoRA Configuration	Rank $r=8$, $\alpha =32$, dropout rate 0.1
	Alternative Models	Qwen
	Fine-tuning Methods	LoRA, Full fine-tuning
	Code Generation	DeepSeek-Chat via API
Hardware	GPU	NVIDIA RTX 3090 (24GB VRAM)
	Memory	128GB DDR4 RAM
Dataset	Source	Code generation tasks
	Training/Testing/Validation	600/200/200 samples
	Libraries	NumPy, Pandas, PyTorch, TensorFlow, Matplotlib
Reward Functions	Overlap	Token overlap between generated and reference code
	ROUGE-L	Longest common subsequence F-measure
	BLEU	*n*-gram precision with smoothing
	F1	Token-based precision and recall
	Code Metrics	Syntax, completeness, and correctness
Training Parameters	Learning Rate	$1\times 10^{-4}$ (Adam)
	Gradient Accumulation	Configurable steps (default: 1)
	Training Epochs	Early stopping applied
Evaluation Metrics	CSS	Code semantic similarity
	Precision	Generation accuracy
	Recall	Reference coverage
	F1 Score	Harmonic mean of precision/recall

We designed experiments on DS-1000 (800 train, 200 test) to answer three research questions. RQ1 compares reward functions (Overlap, ROUGE-L, BLEU-4, F1); RQ2 contrasts LoRA with full fine-tuning; RQ3 evaluates multiple model architectures Qwen-1.8B-Chat, Qwen1.5-0.5B, 1.8B, 4B-Chat, Qwen2-0.5B, 1.5B-Instruct, and Qwen2.5-0.5B, 1.5B, 3B-Instruct. All runs use NVIDIA 3090 GPUs, Adam (lr=1e-4), gradient accumulation (4 steps), and 10 epochs. Table 2 details the complete configuration.

### Evaluation metrics

These metrics are to report the performance of the model. The Code Similarity Score (CSS) measures the sequence correspondence between the generated code *c* and the reference $c^*$:

16 $$\begin{aligned} \textrm{CSS}(c,c^*) = \operatorname {SequenceMatchRatio}(T_c, T_{c^*}) \in [0,1]. \end{aligned}$$

Token sequences are obtained by a simple tokenizer:

17 $$\begin{aligned} T_x = \operatorname {tokenize}(x) = \texttt {re.findall(``[A-Za-z\_]+'' , x)}. \end{aligned}$$

The sequence match ratio normalizes the longest common (contiguous) token subsequence:

18 $$\begin{aligned} \operatorname {SequenceMatchRatio}(T_c,T_{c^*}) = \frac{\operatorname {LCSegLen}(T_c,T_{c^*})}{\max \bigl (|T_c|,\;|T_{c^*}|\bigr )}. \end{aligned}$$

Token-level metrics assess overlap regardless of order:

19 $$\begin{aligned} \textrm{Precision}= & \frac{|\,T_c \cap T_{c^*}\,|}{|\,T_c\,|}. \end{aligned}$$

20 $$\begin{aligned} \textrm{Recall}= & \frac{|\,T_c \cap T_{c^*}\,|}{|\,T_{c^*}\,|}. \end{aligned}$$

21 $$\begin{aligned} \textrm{F1}= & \frac{2 \cdot \textrm{Precision} \cdot \textrm{Recall}}{\textrm{Precision} + \textrm{Recall}}. \end{aligned}$$

All metrics are in [0, 1]. CSS reflects structural similarity via longest contiguous matches, while Precision/Recall/F1 capture exact token overlap.

### Reproducibility

We ensure reproducibility with fixed seeds for Python/NumPy/PyTorch and deterministic flags where available; scripts and configs are released. All reported tables (e.g., Tables 4, 5, 6) use a disclosed fixed seed in captions, and multi-seed runs can be reproduced with the provided configurations. Execution-based evaluation follows a sandboxed harness (2 s per test, safe handling of syntax errors, and timeouts) and reports the unit test pass rate. For external benchmarking, we use the MBPP-lite split under the same generator and decoding setup and metrics (CSS, Precision, Recall, F1), with the complete harness and configurations released for verification.

### Experimental analysis

#### RQ1: Which reward function design performs best for query enhancement?

**Figure 3:**
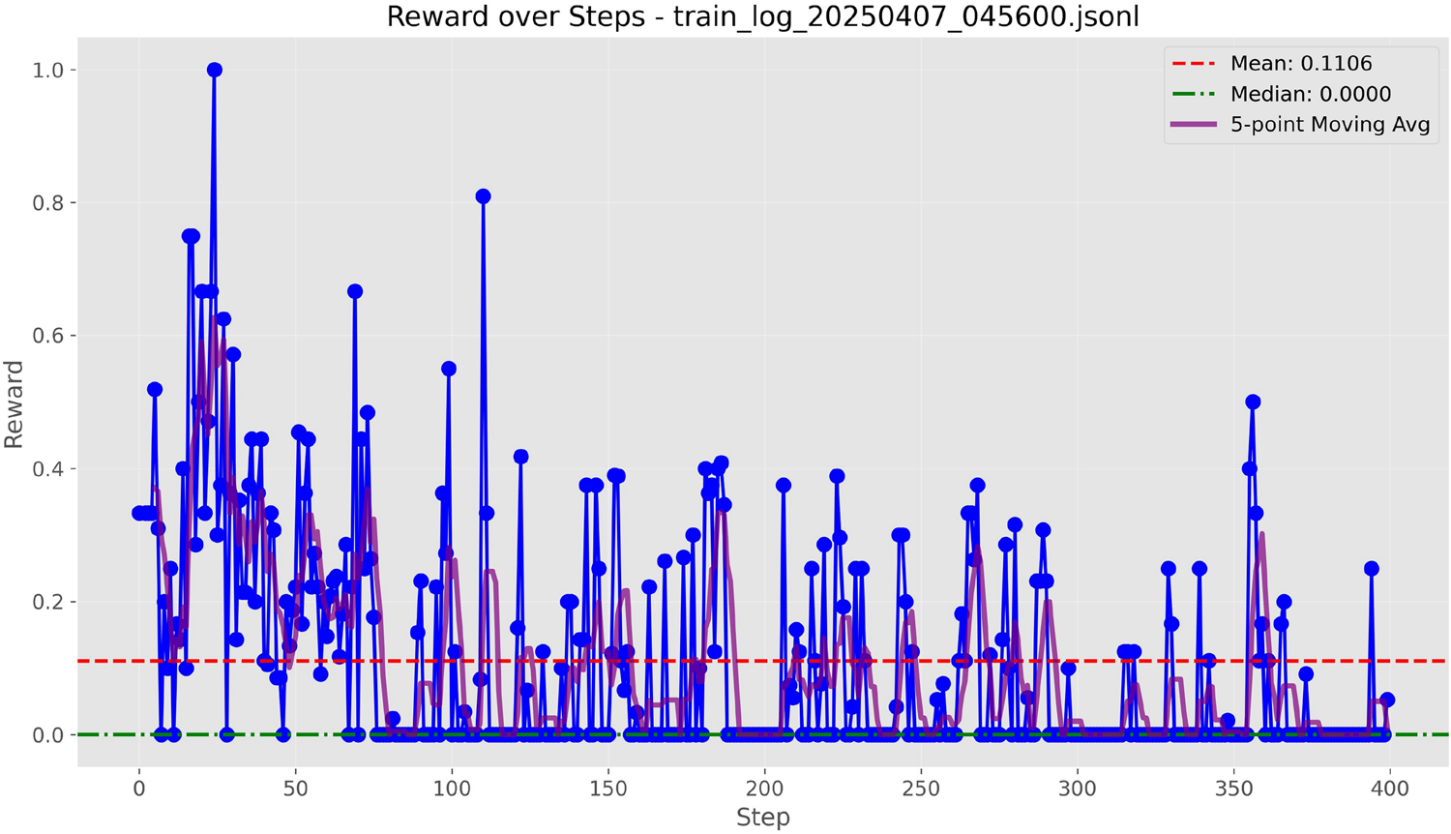
Training curves of Qwen-7B with LoRA ($r=8$) under the Token Overlap metric.

**Figure 4:**
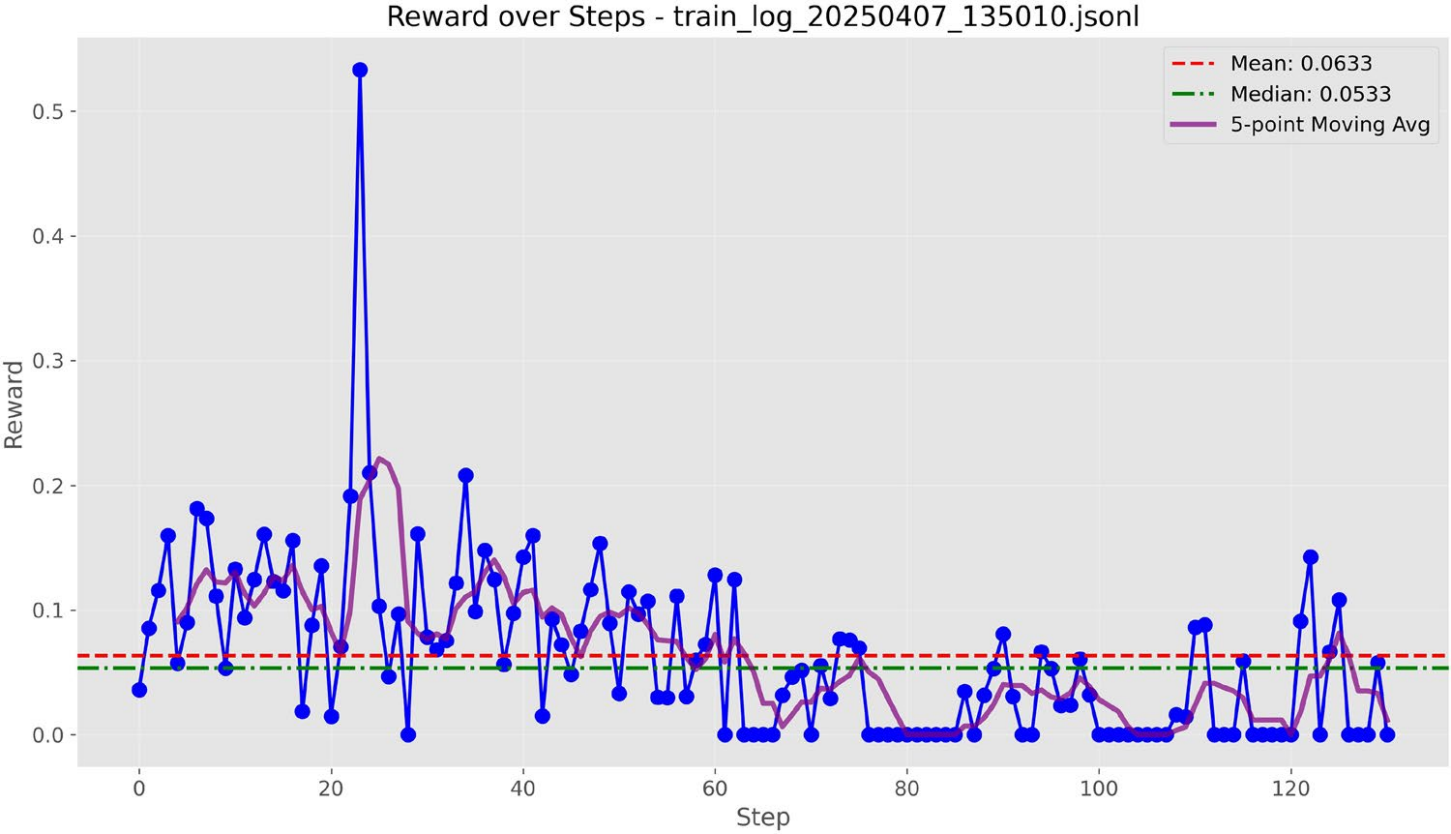
Training curves of Qwen-7B with LoRA ($r=8$) under the ROUGE-L score.

**Figure 5:**
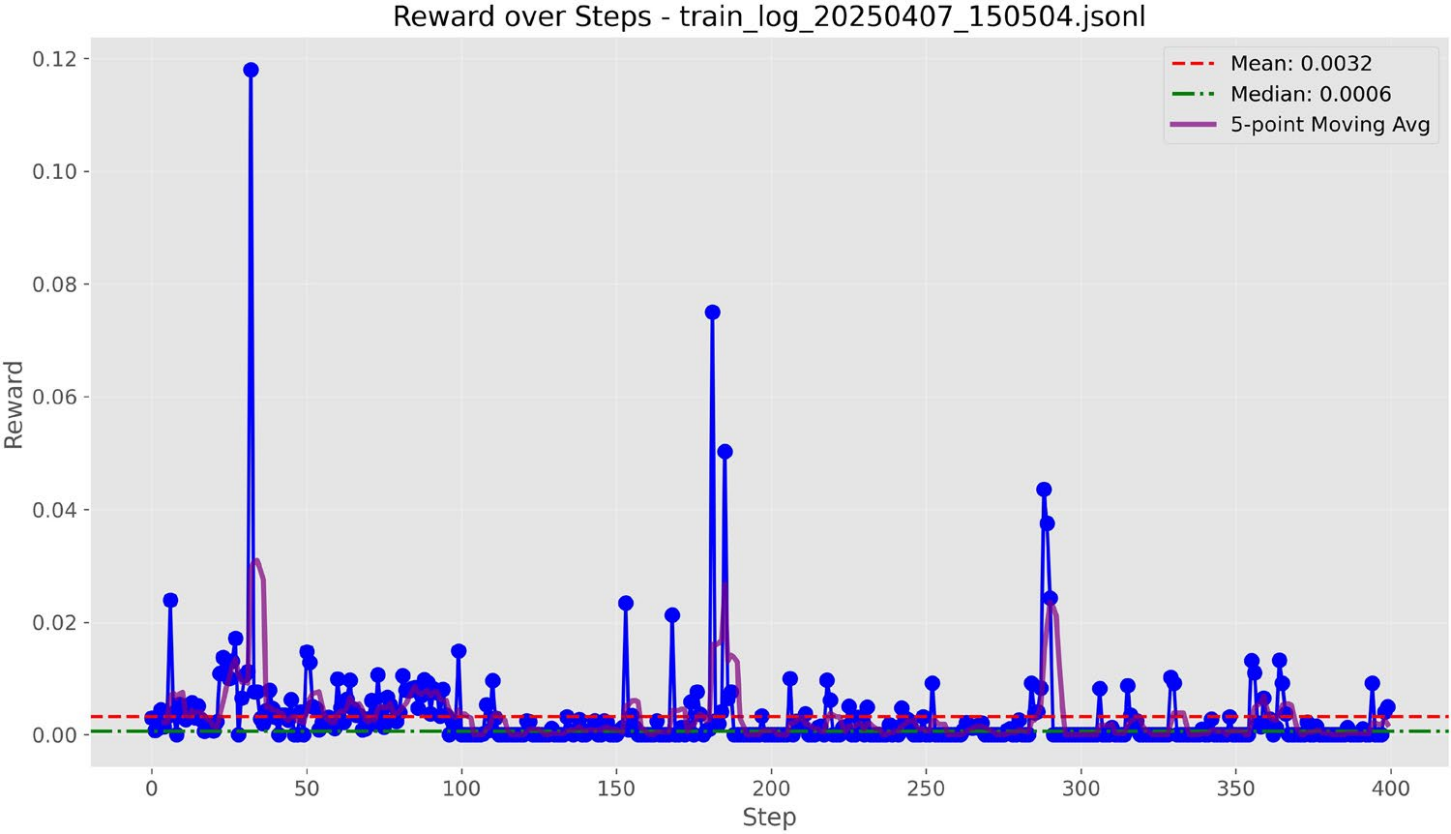
Training curves of Qwen-7B with LoRA ($r=8$) under the BLEU-4 score.

**Figure 6:**
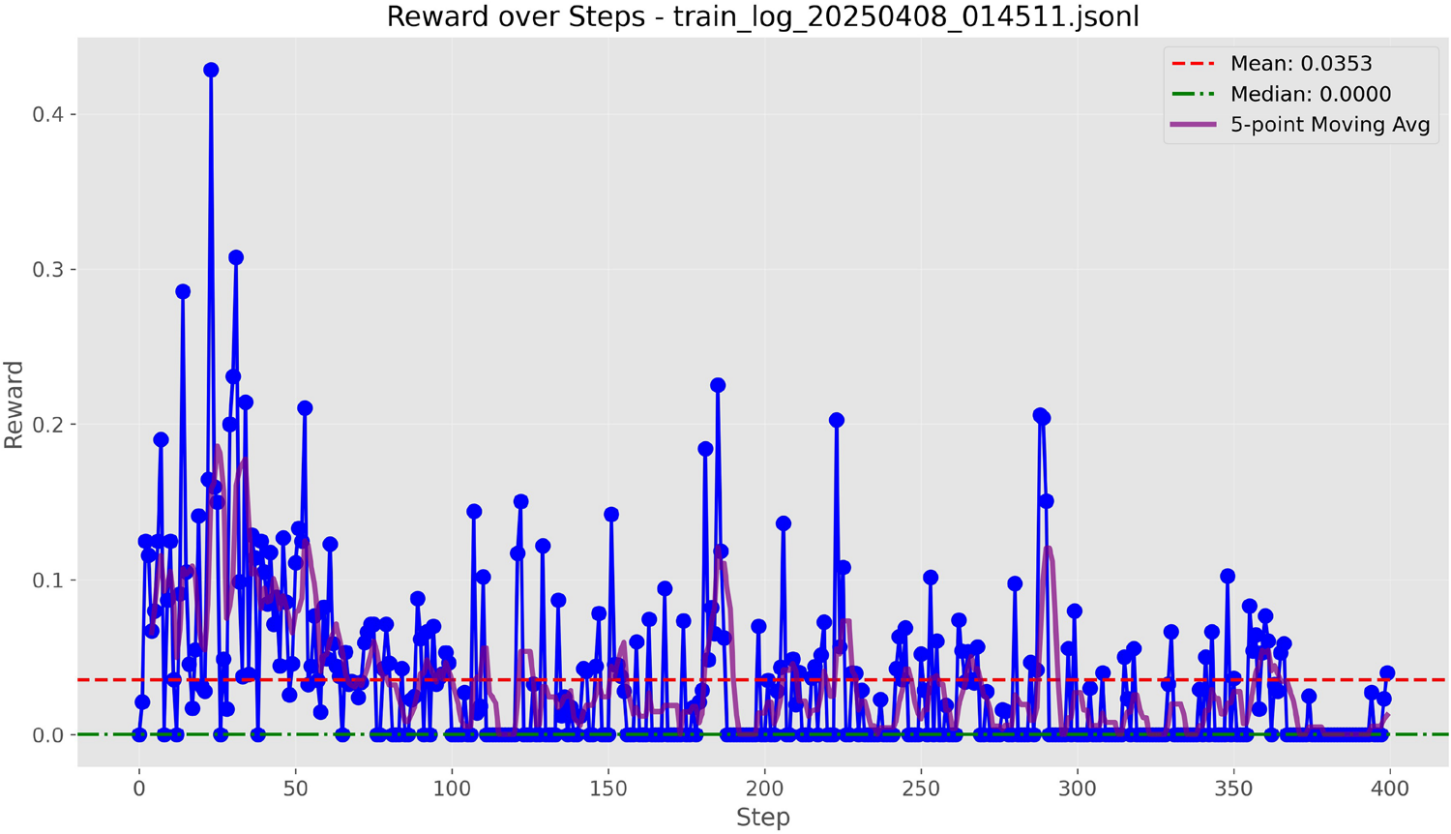
Training curves of Qwen-7B with LoRA ($r=8$) under the F1 score.

**Table 3:** LoRA hyperparameter configuration for Qwen-7B.

Parameter	Value
LoRA rank (*r*)	8 (recommended $r \in [4,64]$ for 7B models)
LoRA alpha ($\alpha$)	32 (scaling factor $\alpha /r=4$)
Dropout rate	0.1 (applied to LoRA weights)
Bias training	Disabled (none)
Target modules	c_attn, c_proj, w1, w2
Precision	FP16 (half precision)
Quantization	8-bit (LLM.int8() scheme)

We evaluated four text evaluation metrics: Overlap, ROUGE-L, BLEU-4, and F1 using the LoRA configuration provided in Table 3. As evidenced by the training curves depicted in Figs. 3, 4, 5, and 6, BLEU-4 consistently produces the most stable and overall rewards. For the Qwen-7B model, which was trained on a comprehensive dataset of 100 samples, the F1 score slightly exceeds BLEU-4. However, when using the smaller Qwen-1.8B model, trained with a more limited dataset of 10 samples, BLEU-4 shows greater robustness. Therefore, we propose BLEU-4 as the default reward metric, recommending F1 as a viable alternative for applications involving larger datasets and models.
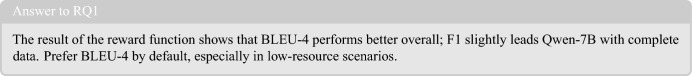


#### RQ2: How does LoRA fine-tuning compare to full fine-tuning?

**Table 4:** Performance comparison of reward metrics on Qwen models with LoRA.

Reward Metric		CSS	Precision	Recall	F1
Overlap	Qwen-7B (r=8, $\rho$=1.0)	–	–	–	–
	Qwen-1.8B (r=8, $\rho$=0.1)	0.0812	0.1173	0.1859	0.1352
ROUGE-L	Qwen-7B	–	–	–	–
	Qwen-1.8B	0.0695	0.0855	0.1950	0.1116
BLEU-4	Qwen-7B	0.0965	0.1250	0.2770	0.1530
	Qwen-1.8B	0.1398	0.1471	0.5608	0.2120
F1	Qwen-7B	0.1016	0.1220	0.2830	0.1510
	Qwen-1.8B	0.0625	0.1107	0.2197	0.1404

**Table 5:** Full fine-tuning vs LoRA on Qwen-1.8B under BLEU-based reward function.

Method	CSS	Precision	Recall	F1
Full Finetuning (Qwen-1.8B)	0.1078	0.1644	0.2028	0.1635
LoRA (Qwen-1.8B, $r=4$)	0.0850	0.1565	0.2054	0.1634
LoRA (Qwen-1.8B, $r=8$)	0.1398	0.1471	0.5608	0.2120
LoRA (Qwen-1.8B, $r=16$)	0.1091	0.1376	0.5046	0.1988

We compare LoRA with full fine-tuning using the results in Tables 4 and 5. Table 4 (LoRA only) shows that, in Qwen-1.8B, BLEU-4 produces the strongest overall metrics (CSS *0.1398*, Precision *0.1471*, Recall *0.5608*, F1 *0.2120*). Table 5 directly contrasts the methods under the BLEU-based reward: LoRA with $r=8$ surpasses full fine-tuning in CSS (*0.1398* vs. *0.1078*), Recall (*0.5608* vs. *0.2028*), and F1 (*0.2120* vs. *0.1635*), while full fine-tuning has the highest precision (*0.1644*). The rank $r=4$ is essentially on par with full fine tuning in F1 (*0.1634* vs. *0.1635*), and $r=16$ improves over full fine tuning but underperforms $r=8$ (F1 *0.1988* vs. *0.2120*), indicating diminishing returns beyond $r=8$.
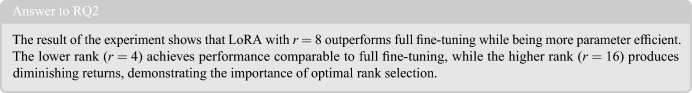


#### RQ3: How does our approach perform on different foundation models?

**Table 6:** Comparative analysis of foundation models with LoRA ($r=8$, $\rho =0.1$).

Model variant	Precision	Recall	F1	CSS
Qwen-1.8B-Chat	0.0969	0.2677	0.1121	0.0799
Qwen1.5-0.5B-Chat	0.0972	0.1908	0.1166	0.0660
Qwen1.5-1.8B-Chat	0.2301	0.6590	0.3264	0.2154
Qwen1.5-4B-Chat	0.1692	0.3365	0.2091	0.1125
Qwen2-0.5B-Instruct	0.1778	0.7240	0.2704	0.1204
Qwen2-1.5B-Instruct	0.1769	0.7699	0.2778	0.1455
Qwen2.5-0.5B-Instruct	0.2041	0.7490	0.3070	0.1949
Qwen2.5-1.5B-Instruct	0.1501	0.7492	0.2408	0.1152
Qwen2.5-3B-Instruct	0.2250	0.7088	0.3193	0.1956

We evaluated our approach in all foundation models with LoRA ($r=8$, $\rho =0.1$). Table 6 shows that architecture matters more than size: Qwen1.5-1.8B-Chat achieves the best balance (CSS *0.2154*, F1 *0.3264*), outperforming larger models (e.g., Qwen1.5-4B-Chat, Qwen2.5-3B-Instruct). The Qwen2 / 2.5 variants produce high recall (up to *0.7699* for Qwen2-1.5B-Instruct) but less balanced precision / CSS, while the Qwen1.5 series is more consistent, especially the 1.8B model (metrics scaled to [0,1]; bold denotes the best column in Table 6).
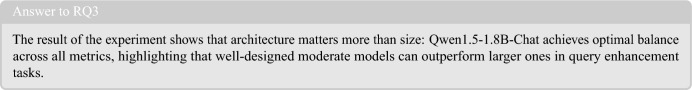


## Discussion

Reinforcement learning improves code generation by refining queries^42,44,45^, helping to close the *query gap* between natural language and effective prompts^41,46,47^. It turns vague inputs into precise technical prompts^48–50^, taking advantage of established prompting and feedback patterns^43,51^. A multi-aspect reward is beneficial^52–54^, while LoRA maintains efficiency with performance comparable to full fine-tuning^40,55,56^. Differences between model families highlight the importance of code-pre-trained foundations^13,57^. Recent advances in RL offer practical extensions: attention-prioritized replay for sample efficiency^59^ and weighted mean field Q-learning for stable aggregation of enhanced queries^60^. Empirically, rank $r{=}8$ balances capacity, stability, and compute: lower ranks underfit attention projections; higher ranks raise parameters and variance, yielding diminishing returns. Limitations include DS1000 domain and horizon coverage^5,7,8^ and generator sensitivity^10,11,28^. Human feedback can further improve efficiency and reward design^9,14,25^. Future work will explore more efficient RL^16,19,20^, hybrid strategies^22–24^, broader domains^26,27,29^, personalization^30–32^, and interactive systems^33,35,36^ to make AI assistance more accessible across skill levels^12,37–39^. Moreover, we introduced RL4QE, a practical framework that learns to refine queries with a parametric refiner (Qwen+LoRA) while keeping the code generator (DeepSeek) fixed. Across DS-1000 experiments, BLEU-4 emerges as a strong default reward (F1 competitive on a larger scale), and LoRA with $r{=}8$ outperforms full fine-tuning on most metrics while using fewer trainable parameters. The approach is transferable across foundation models and is easy to integrate (LoRA on attention projections). We release code, seeds, and harnesses to support reproducibility and external verification.

## Data Availability

The datasets and code used in this study are publicly available at: https://github.com/davidyuan666/RL4QE and 10.6084/m9.figshare.28767299.v2. The DS1000 used for the evaluation can be accessed from the original repository.
